# Investigating Topic Modeling Techniques to Extract Meaningful Insights in Italian Long COVID Narration

**DOI:** 10.3390/biotech11030041

**Published:** 2022-09-03

**Authors:** Ileana Scarpino, Chiara Zucco, Rosarina Vallelunga, Francesco Luzza, Mario Cannataro

**Affiliations:** 1 Department of Medical and Surgical Sciences, University “Magna Græcia”, 88100 Catanzaro, Italy; 2 Data Analytics Research Center, University “Magna Græcia”, 88100 Catanzaro, Italy; 3 Department of Health Sciences, University “Magna Græcia”, 88100 Catanzaro, Italy

**Keywords:** text mining, topic modeling, LDA, BERTopic, narrative medicine

## Abstract

Through an adequate survey of the history of the disease, Narrative Medicine (NM) aims to allow the definition and implementation of an effective, appropriate, and shared treatment path. In the present study different topic modeling techniques are compared, as Latent Dirichlet Allocation (LDA) and topic modeling based on BERT transformer, to extract meaningful insights in the Italian narration of COVID-19 pandemic. In particular, the main focus was the characterization of Post-acute Sequelae of COVID-19, (i.e., PASC) writings as opposed to writings by health professionals and general reflections on COVID-19, (i.e., non-PASC) writings, modeled as a semi-supervised task. The results show that the BERTopic-based approach outperforms the LDA-base approach by grouping in the same cluster the 97.26% of analyzed documents, and reaching an overall accuracy of 91.97%.

## 1. Introduction

Among the effect of the COVID-19 pandemic, an unprecedented widespread and severe impoverishment of mental health among people has been registered [1,2,3]. Through the massive use of social networks, registered during periods of social isolation and lockdown restrictions, people have often narrated their experience in dealing with the pandemic, sharing their physical and emotional state.

The large amount of data available for collection and analysis has therefore allowed the scientific community to apply text mining techniques to summarize and investigate various aspects related to the narrative of the COVID-19 pandemic.

Several studies have focused on extracting topics in tweets related to the COVID-19 pandemic [4,5,6]. In particular, the study of Medford et al. [7] collected tweets related to COVID-19 in January 2020, during the initial phase of the outbreak, measuring the themes and frequency of keywords related to infection prevention practices, identifying the polarity of sentiment and predominant emotions in the tweeting and conducting a topic modeling of the topics of discussion over time through LDA.

Valdez et al. [8] applied the LDA model and sentiment analysis techniques [9] for the identification of topics and the trend of sentiment in Twitter users about the pandemic. The authors’ conclusions assert that the use of the social network has allowed activating sometimes coping mechanism to combat feelings of isolation related to long-term social distancing [8] and sometimes exacerbating concern and disinformation [10]. Also LDA was exploited by Jelodar et al. [11] and Leun et al. [12] to identify topics and understand the concerns of Reddit and Twitter users respectively.

The evaluation of the topics most discussed by people who have faced the disease from COVID-19 can lead to useful applications in the clinical setting, however it also poses several challenges. In particular, the analysis of tweets is limited in the number of words the user can post. Secondly, the ability to collect tweets by hashtags or keywords search on the one hand allows the researcher to collect a lot of relevant data in a short time, but on the other hand it makes it difficult to distinguish in a massive collection of tweets the ones that are posted by users who have actually contracted COVID-19 disease.

In our view, an important contribution can be made by the automatic analysis of Narrative Medicine textual content. Narrative Medicine (NM) is a practice that provides tools for listening, support and comfort to the patient.

NM integrates with Evidence-Based Medicine (EBM) to lead to clinical care decisions which are more complete, personalized, effective, and appropriate, taking into account the plurality of perspectives [13]. In a synergistic perspective, the narration of patient and caregivers should play an essential role in contemporary medicine [14].

Of particular interest are the narratives of patients with Post-acute Sequelae of COVID-19 (PASC), also known as Long COVID syndrome. Post-acute Sequelae of COVID-19, also known as “post-COVID-19 syndrome” or “long-haul COVID”, refers to those convalescent individuals in whom prolonged and often debilitating sequelae persist [15]. The clinical symptomatology includes several main manifestations, often affecting different organ systems. In addition to fatigue, malaise and dyspnoea, PASC patients may also be affected by a number of psychiatric disorders, including depression, anxiety, and post-traumatic stress disorder [16].

In Pye et al. [17] a prevalence estimate was conducted to identify PASC and predict fluctuations in the number of people experiencing persistent symptoms over time.

In Baum et al. [18,19] Hidden Markov Models (HMM) were applied to speech and text recognition. The HMM is a popular statistical tool for modelling a wide range of time series data. In the context of NLP, HMMs have been applied with great success to problems such as part-of-speech tagging, noun-phrase chunking and extracting target information from documents [20].

In another study [13], evidence of mood and cognitive impairment was found that urgently needs the development of targeted therapies and the support of telemedicine.

By extending the work in [21,22], the present paper focuses on applying Natural Language Processing (NLP) and Text Mining (TM) methods, and in particular different topic modeling techniques, to extract useful insights from Narrative Medicine textual sources to characterize the writings of PASC from narratives of subjects who haven’t experienced the PASC condition, as for instance patient’s relatives or healthcare professionals.

To this extent, we automatically collected 187 Italian NM textual testimonies encompassing the narration of PASC patients, healthcare professionals, and general reflections about the COVID-19 disease. The automatic collection was performed through web scraping of two different NM Italian blogs, i.e., “Sindrome Post COVID-19” (https://www.sindromepostCOVID-19.it/, accessed on 30 August 2022) and “R-Esistere” (https://www.medicinanarrativa.network/r-esistere/, accessed on 30 August 2022).

More in details, 73 among the 187 textual narrations were blog posts from “Sindrome Post COVID-19”, an Italian blog collecting testimonies of those who have had or are facing the PASC syndrome, while the remaining 114 texts were blog posts from “R-Esistere”, another Italian blog site hosting testimonies of illness, treatments, healing or loss, but also the narration of healthcare professionals and citizens wanting to share reflections about the pandemic.

Narrations collected from “Sindrome Post COVID-19” were tagged as “PASC” texts, while narrations collected from “R-Esistere” were tagged as “Non-PASC” testimonies. The automatic tagging process was followed by a manual tagging phase, to verify all the writings.

Among the existing topic modeling techniques, two different approaches were explored, i.e., Latent Dirichlet Allocation, which is one of the most common approaches, and another approach based on the popular BERT Transformer [23]. Transformer is a prominent deep learning model that has been widely adopted in various fields, such as NLP, computer vision (CV) and speech processing. Transformer architecture can be used in different ways as Encoder-Decoder. This is typically used in sequence-to-sequence modeling (e.g., neural machine translation) [24]. Since the goal is to create a model that performs a number of different NLP tasks, Bert is used in this approach.

The rest of the paper is organized as follows. In Section 2 the background information about NM and TM is introduced, and an overview of topic modeling application on COVID-19 related text is discussed. Section 3 describes the data collection, the dataset and the methodology behind the proposed application and Section 4 discussed the result of the analysis. Finally, Section 5 concludes the paper and outlines future works.

## 2. Background

Medicine practiced with narrative competence may be better able to recognize patients and diseases, empathize with colleagues, accompany patients and their families through the vicissitudes of the disease [25,26,27,28]. In particular, NM has many advantages: it improves clinical practice, allows a more in-depth diagnosis, promotes adherence to therapy, helps and consolidates choices, fosters relationships between patient, family, and healthcare staff, improves the quality of service and the therapy strategy, verifies and allows feedback on the functionality of the therapy, promotes the formation of communities that help the patient on a social and psychological level.

The methodologies that have characterized the analysis of written and oral narrative material can be grouped into three main strands: Thematic Analysis allows to count the frequency of the words and themes proposed by the patient [29]; Linguistic Analysis allows to differentiate the narratives by gender complexity [30]; Content analysis implements various procedures for a quantitative survey of the narrative structure and its qualitative content [31]. In the context of COVID-19 support groups have emerged that illustrate the rhetorical nature of the narratives. Specifically, in Long Covid, the characteristic of the narration is that it occurs almost exclusively among patients. Long Covid calls for socio-narratological examination as a novel illness where both trust (on the patients’ part) and obligation (on their doctors’) went awry. Such a target, moreover, may help explore the metanarrative twist in Long Covid’s own story—that doctors and healthcare workers were disproportionately affected since COVID-19 is an occupational disease. Long Covid was not only a new illness but one which, perhaps uniquely in recent history, emerged largely without the patient’s clinician acting as a witness or sounding board. The story of Long Covid as it unfolded in 2020 includes an overarching metanarrative of absent listeners: the collective failure—arguably for good reasons—of clinicians to acknowledge, interpret or act on their patients’ stories and plights [32].

In the context of the emergency from COVID-19 the Autoregressive Integrated Moving Average (ARIMA) time series (TS) analysis model was employed to analyze the temporal dynamics of the worldwide spread of COVID-19 in the time window from 22 January 2020 to 7 April 2020 [33].

In Zhou et al. [34] the authors applied an overall HMM that, based on multiple nations’ COVID-19 data including the USA, several European countries, and other countries that have strict control policies, explore different types of observations. They used it to infer the severity of disease on small geographical states or regions in the USA and Italy as test cases.

In Prabhu et al. [35] Hidden Markov Models have been applied to get a better assessment of the extent of spread.

### 2.1. Text Mining

TM is a branch of NLP that deals in particular with the automatic extraction of unknown information from textual inputs. It uses Data Mining techniques to identify information that would otherwise remain locked up in a mass of unstructured textual data. Subsequently, the information, once extracted, is transformed into structured data thanks to the use of NLP algorithms, which thus read and analyze textual information, recognizing similar concepts, even if they are expressed in different ways. To prepare the text data for the analysis, text preprocessing is performed. Some of the preprocessing steps are: removing punctuations; removing URLs; removing stop words; lower casing; tokenization; stemming; lemmatization. The aim of the research concerning TM is therefore to study new methods and algorithms to automatically extract knowledge from the text, for example, to classify or group documents based on content. In TM there is more possibility of using indexing, which is useful for indexing and counting words so that the frequency matrix is constructed, i.e., a table that enumerates the occurrence of each word in each document. It is thus possible to apply the various statistics to obtain the keywords that determine the most important words from a text. While data mining can be freely defined as the search for patterns in the data (typically numeric), TM is the search for patterns in the text. In this field, NLP operates efficiently on unstructured data which is transformed into structured data through appropriate algorithms [36]. Other applications of TM include document summarization, entity extraction for identifying the entities or sentiment analysis for the emotion detection from written natural language.

### 2.2. Topic Extraction on COVID-19 Related Texts

COVID-19 has had an impact on various aspects of individual well-being enough to lead people to express different opinions on various topics related to the new virus. We are often faced with a lot of text data on social media or research papers. There is a hidden theme structure in every text data. TM analysis focuses on this. The significance of the technique deployed, namely LDA, can be gauged from its name itself: “Latent” means hidden. Here we are talking about undercurrent themes or topics which are concealed. Dirichlet is based on the concept of “distribution of distributions” [37]. Various studies incorporated text mining techniques such as topic modeling analysis or LDA [38,39] to identify and visualize the topics and patterns of COVID-19 literature. However, these studies either had a larger focus on all coronavirus infection types, including Post-acute COVID-19 syndrome [40]. Therefore TM methods aim to obtain information from documents and this application to the COVID-19 literature is new. Researchers from around the world are trying to understand various aspects of the COVID-19 pandemic. Since the identification of the COVID-19 disease in December 2019, numerous studies from various fields have already been published. NLP based research that analyzes COVID-19 related material such as scientific articles, social media posts, and news is one example of the growing body of research related to COVID-19 [41]. Bai et al. [42] present a topic evolution analysis of COVID-19 news articles from Canada. Liu et al. [43] used a digital topic modeling approach to analyze news media during the early stage of the COVID-19 outbreak in China. De Santis et al. [44] presented an infoveillance system for detecting and tracking relevant topics from Italian tweets during the COVID-19 event. Noor et al. [45] analyzed the public reactions to the novel COVID-19 outbreak on Twitter. The type of TM study conducted by Han et al. [46] helped the government in understanding public opinion regarding COVID-19 to better support public assistance during the pandemic. Han et al. [46] used social media posts from China during the first COVID-19 outbreak to estimate public opinion regarding the disease. They used LDA to categorize posts into seven topics, examining the relationship between social media post topics and the intensity of the outbreak in parts of China. The results of this study showed that the timely release of information by the Chinese government helped calm public opinion in the early stages of COVID-19. Nguyen [47] provides a recent review of TM and NLP in the context of COVID-19, on several examples of how researchers use Twitter’s data mining applications to get predictions on infection rate and to better understand the changing government policies and responses to COVID-19 [48]. There are significant limitations concerning this kind of analysis, as the low availability of the works analyzing italian texts, NM texts in general and which use Transformers architecture, like Bert. Another limit is that the majority of these studies use data Twitter that are short but in large quantity.

## 3. Materials and Methods

Aim of the present study is to apply standard NLP techniques, and in particular two different topic modeling approaches, for the analysis of italian texts written about the COVID-19 disease belonging to two different categories: texts written by subject with post-COVID disease (PASC), and writings of healthcare professional and general reflections by citizens (Non-PASC), with the aim of assessing whether it is possible to characterize PASC patients textual narration by discussed topics. Both data collection and data analysis were performed in Python, a high-level programming language.

### 3.1. The Italian COVID-19 Narrative Medicine Dataset

For the present study, 187 narrations related to COVID-19 have been automatically collected from two Italian projects: “R-Esistere” (https://www.medicinanarrativa.network/r-esistere/, accessed on 30 August 2022) and “Sindrome post COVID-19” (https://www.sindromepostcovid19.it/, accessed on 30 August 2022) and grouped in PASC and non-PASC testimonies.

To the best of our knowledge, the Italian project Sindrome Post COVID-19 can be considered as the first attempt to create a database that stores the testimonies of those who have had, or are currently facing, the PASC syndrome. To ensure reliable and high-quality data, people who shared their experiences have been required to provide their social security number along with specific information about their symptoms after test negativization. All the narrations shared on the Sindrome Post Covid project’s blog, i.e., 73 testimonies, were automatically collected and classified as PASC texts.

The remaining 114 texts have been automatically collected from the Italian blog “R-Esistere”. R-Existere is a project developed by the Italian Society of Narrative Medicine (https://www.medicinanarrativa.network/, accessed on 30 August 2022), which collects stories of patients, relatives, citizens and health professionals in order to make citizens feel understood, but also to make available data useful for scientific research and understand and review critical issues, protocols and health organizations related to the pandemic. The textual testimonies collected from the R-esistere blog, were classified as “non-PASC” category. All the narrations were manually rechecked to confirm the automatic categorization.

Python’s “Beautiful Soap” and “requests” libraries have been used to automatically collect the NM testimonies and to store the data in a single CSV file.

For instance, to collect data from the “Sindrome post COVID-19” blog, the Python code:

- Collects all the URL of the testimonies per page,
- connects to the page and, by parsing the HTML document:
  - extracts only the textual narration,
  - performs a basic text cleaning,
  - tags it as “PASC”,
- the extracted information is stored in a CSV file format.

The described process is then iterated through all the narrations posted on the blog for both the considered blogs. The information is stored in a *CSV* format file for the subsequent analysis and integrated with the data collected from the “R-esistere” blog following a similar data collection pipeline.

For each considered blog, the CSV stores data sorted by chronological order: from the most recent to the oldest post.

### 3.2. Topic Modeling Using LDA

A topic can be seen as a collection of representative words in a text, that help to identify what is the subject or the subjects of a document.

Latent Dirichlet Allocation (LDA) [49] is a generative probabilistic model commonly used for the identification of latent topic in textual corpora.

The starting point of an LDA model is that a document is represented as a bag-of-words. Each single word is represented as a pair of values: the first represent its position, the second is the number of occurrences of the word itself within the document. The assumption under an LDA model is that each document in a corpus can be modeled as a mixture of a finite number of topics with a certain probability, while a topic can be characterized by a distribution over words.

More in details, assume that there are *k* topics across all documents. Let w be a document in a corpus D and let consider:

- θ∼Dir(α), be a mixture of *k* topics, having a Dirichlet probability distribution where α is the per-document topic distribution;
- a topic $z_{n}∼Multinomial\left(\theta \right)$, where *n* represents the number of words that define a topic;

then, given the topic $z_{n}$, a word $w_{n}$ is sampled from $p(w_{n}|z_{n},\beta )$, and β represents the per-topic word distribution. Then, the probability of w containing *n* words can be described as (Equation (1)):

(1) $$p\left(w\right)=\int \left(\prod_{n=1}^{N}\sum_{z_{n}=1}^{k}p\left(w_{n}|z_{n},\beta \right)\right)p\left(\theta ,\alpha \right)d\theta$$

The implementation of LDA used in this paper is the one provided by the gensim [50] Python library.

### 3.3. Topic Modeling Using BERT

Although Bag-of-words is a classic technique of representing textual documents, one of its limitations lies in the fact that it does not take into account the context in which the word is inserted in a sentence. Consequently, the Bag-of-words approach does not capture semantic relationships between words.

With advancements in the Deep Learning field, word embedding techniques have become state-of-the-art in recent years. More specifically, the most recent representation models use architectures known as transformers [51], the most popular of which is certainly Bidirectional Encoder Representations from Transformers (BERT) [23] with its variants, as for instance RoBERTa [52], DistilBERT [53], DistilUSE etc.

Word embedding techniques have proved useful in several Natural Language Processing tasks. Regarding topic modeling, the basic idea is to approach topic modeling as a cluster embedding task.

Among the various proposed techniques, this work will focus on BERTopic. BERTopic generates topic representations through a four steps process: it converts documents to their embedding representation using a pre-trained language model, it performs a dimensionality reduction through UMAP [54], it leverages HDBSCAN algorithm [55] to cluster texts in groups that have a similar meaning, then it uses a class-based variation of Term Frequency- Inverse Document Frequency (TF-IDF) (TF-IDF is a common weighting scheme that aims to give a vector representation of each document that reflects the importance of each word by calculating the normalized product of the Term Frequency and the Inverse Document Frequency, which depends on the inverse fraction of the documents that contain the word.) to retrieve the most representative words for each cluster/topic.

### 3.4. Data Analysis Pipeline

The pipeline for the analysis of textual data and the extraction of latent topic for the Italian language through an LDA-based approach, follows our previous works [21,22]. However, differently from our previous work [21], different test batteries were performed and two different topic approach were tested. The general analysis pipeline involves the following steps:

- **Text preprocessing:** includes standard NLP (Natural Language Processing) techniques. A key importance is played by text cleaning techniques that encompasses lowercasing, punctuation, separator, special characters and stop words removal. The preprocessing step has been executed by using regular expression, and the popular *nltk* (https://www.nltk.org/, accessed on 30 August 2022) and *SpaCy* (https://spacy.io/, accessed on 30 August 2022) libraries for Natural Language Processing in Python, which provide a set of preprocessing algorithms also for the Italian language. However, the set of stopwords provided by the aforementioned libraries has been massively expanded to improve the perfomance of preprocessing.
- **Feature extraction:** for each document, lemmatization has been carried out to reduce the inflected form of a word in its canonical form called “lemma”. After lemmatization, a word dictionary was built through the implemented function provided by the *gensim* (https://radimrehurek.com/gensim/, accessed on 30 August 2022) Python library [50]. The construction of the dictionary is essential to generate the bag-of-words model, which contains a numeric representation of the input sentences and, as said before, it constitute the starting point textual representation for an LDA approach. Therefore the present step of the pipeline has not been considered when performing topic modeling through a BERTopic-based approach.
- **Topic modeling:** In a first test battery Latent Dirichlet Allocation (LDA) model [49] has been exploited to extract relevant topic. For each document in the dataset, we considered the associated topic as the topic reaching higher probability. To select the LDA models that better fits the data and to assess the model’s clarity, perplexity and coherence score (Perplexity is an intrinsic evaluation method that statistically measures of how well a probability model predicts a sample while topic coherence scores a single topic by measuring the degree of semantic similarity between high scoring words in the topic.) have been considered as evaluation metrics. Both topic coherence and perplexity were calculated through the *gensim*library. In a second test battery topics were modeled with a BERTopic-based approach, using the BERTopic Python library available at https://github.com/MaartenGr/BERTopic, accessed on 30 August 2022. Working with the Italian language, as language model we choose a multilingual sentence-transformers model. Sentence-transformers are a class of language models tuned to be used for sentence embedding generation [56]. In particular, the tested multilingual sentence embedding model was the *distiluse-base-multilingual-cased-v1* model (https://www.sbert.net/docs/pretrained_models.html, accessed on 30 August 2022).

## 4. Results and Discussion

In this section, we present the result of testing two different topic models to assess whether it is possible to characterize PASC patients textual narration by topics.

Moreover, to gain useful insights about the collected data, a preliminary exploratory data analysis was performed by summarizing data through suitable visualization.

### 4.1. Exploratory Data Analysis

**Figure 1:**
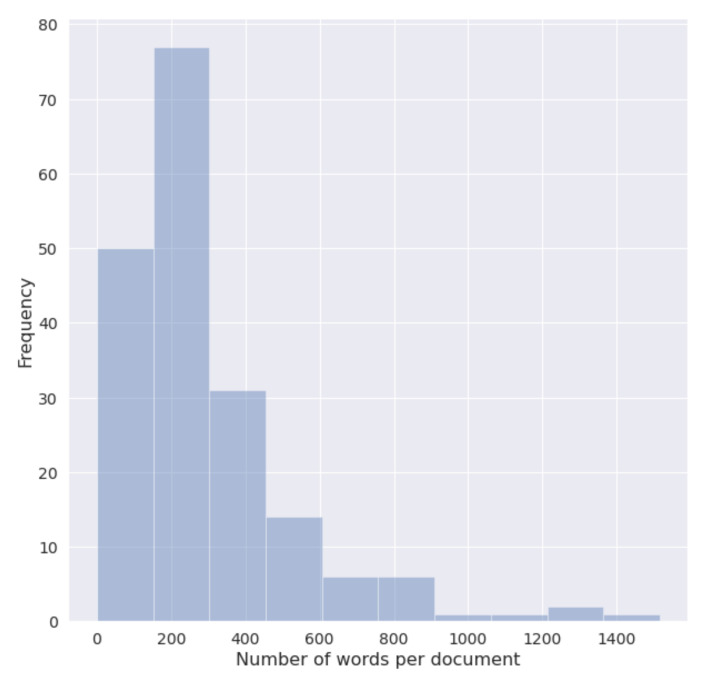
Histogram of words count per document of the entire dataset.

**Figure 2:**
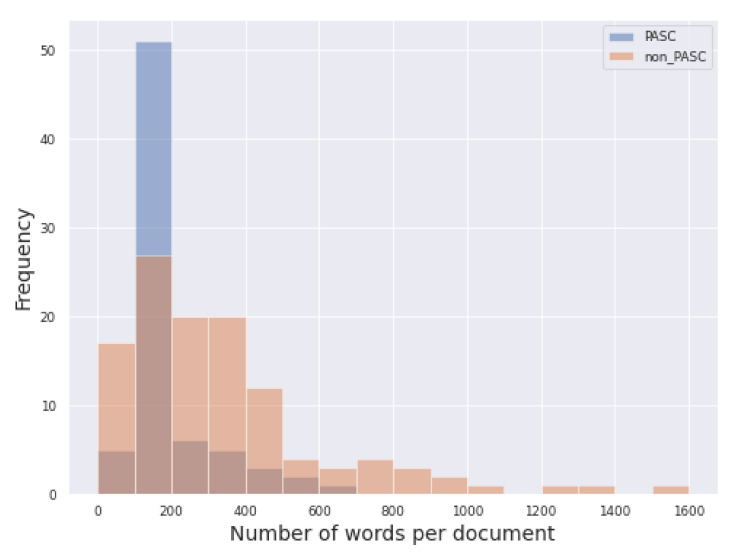
Histogram of words count per document and class.

The shortest document is composed by 21 words, while the longest counts 1516 words, both belonging to the non-PASC category, while the longest document among PASC narrative counts 660 words. On average, the PASC texts tend to be shorter, with a mean of 204.64 word counts, while non-PASC narratives count a mean of 344.64 words. The majority of documents count less than 700 words, and an interquartile range of 366.5 words. Figure 1 and Figure 2 show the distribution of documents in terms of words count for the entire dataset, and for each category, respectively.

**Figure 3:**
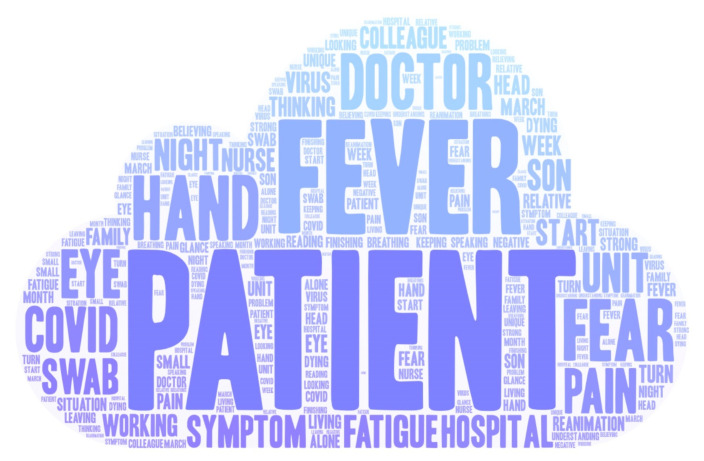
Word cloud showing the most frequent tokens in the document after preprocessing. Tokens with the largest font size are the most frequent.

To further provide some insights about the latent aspects more frequently mentioned in the corpus (see Figure 3), the words more used across the documents are shown through a word cloud. The corpus was pre-processed with standard NLP techniques, i.e., tokenization, stop word removal, and lemmatization and then the word cloud was built by a Python word cloud generator. Terms as “patient”, “fever”, “doctor”, “hand”, “fear”, “pain”, “covid”, “eye”, “swab” are the most frequent words in the corpus, followed by “fatigue”, “hospital” and “symptom”.

### 4.2. Lda Topic Modeling

The topic were extracted from the corpus through the analysis pipeline presented in Section 3.4. As stated before, to select the LDA model that better fits data, the number *k* of topics has been made to vary among 3,5,7 and k=10 topics and the models performance have been evaluated in terms of coherence and perplexity. As shown in Table 1, the best trade-off between the two measures is achieved for k=3.

**Figure 4:**
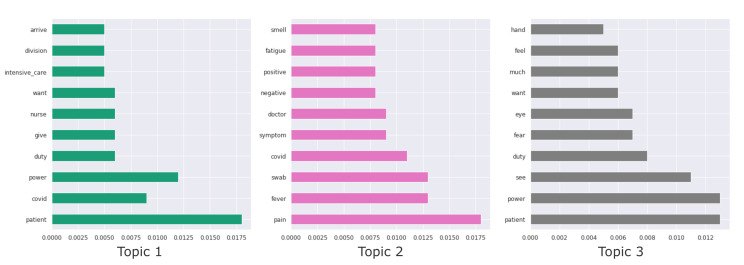
Bar plot of keywords and weight for each topic modeled through the LDA approach.

Topic were considered as a set of 10 keywords. In Figure 4 the topics generated by LDA with k=3 are represented with a bar plot, showing for each keyword the respective weight. Note that reported words have been translated from Italian to English to improve readability (see Figure 3 and Figure 4).

**Figure 5:**
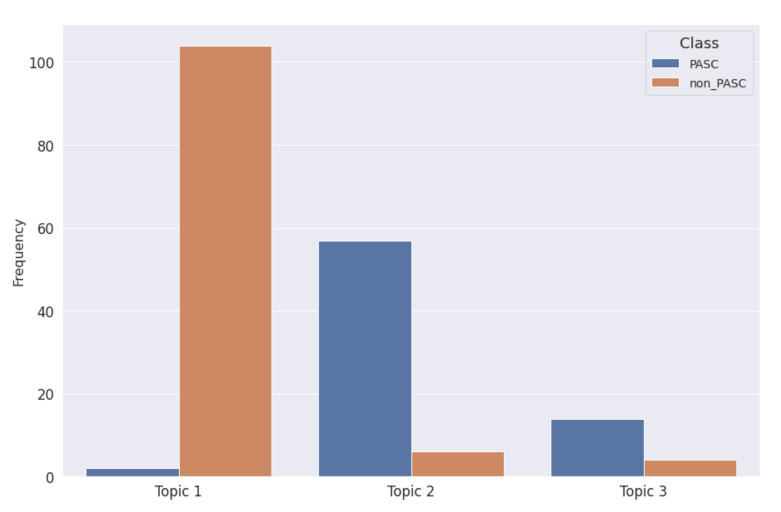
Bar chart describing the distribution of topics modeled trough the LDA approach w.r.t. PASC or non-PASC classes.

The bar chart in Figure 5 shows the distribution of topics per class. From the Figure 5 there is a clear separation of topic 1 and topic 2 in the two classes, while the difference of distribution among the classes is less accentuated.

As previously stated, when a document was assigned to more than one topic, we considered the one with higher probability.

### 4.3. Bertopic-Based Topic Modeling

To extract relevant topic with BERTopic, the analysis pipeline presented in Section 3.4 was followed, with *distiluse-base-multilingual-cased-v1* as multilingual embedding model, and the language set to Italian.

**Figure 6:**
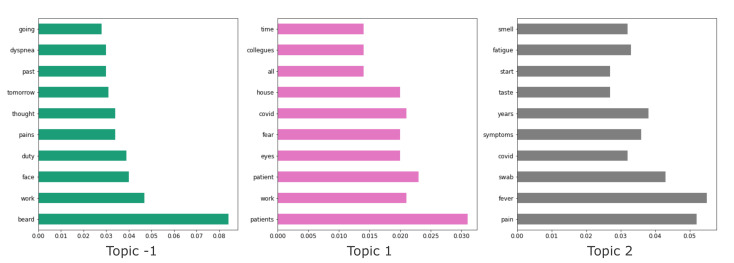
Bar plot of keywords and weight for each topic modeled through the BERTopic approach. Topic -1 represents a virtual topic of unassigned documents to other topics.

As for LDA topic modeling, topics were considered as a set of 10 keywords. In Figure 6 the topics generated by BERTopic are represented with a bar plot, showing for each keyword the respective weight.

**Figure 7:**
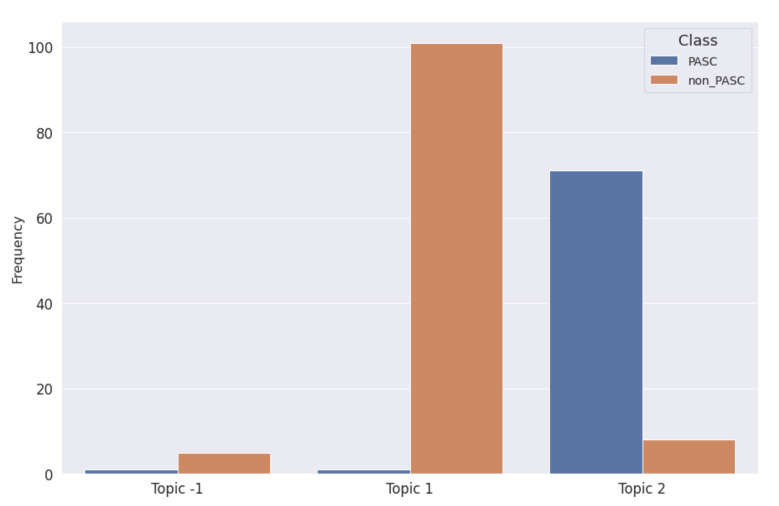
Bar chart describing the distribution of topics modeled trough the BERTopic approach wrt PASC or non-PASC classes.

The bar chart in Figure 7 shows the distribution of topics per class. Note that reported words in Figure 7 have been translated from Italian to English to improve readability.

Unlike the LDA approach, which forces the assignment of all the documents in the corpus to at least one topic, BERTopic considers a cluster of unassigned documents, called “Topic -1”.

The results reported in Figure 7 shows that among the 187 documents, 6 have been unassigned to any topic, 5 belonging to non-PASC class and 1 PASC testimonies.

### 4.4. Comparing BERTopic with LDA for the Characterizazion of PASC Narrative

For a comparative analysis of the topics modeled through LDA and through BERTopic, the problem of characterizing the narrative of PASC patients was considered as a semi-supervised task.

In particular, for each of the two topic model approaches, each document was assigned to the topic with the highest probability.

Consequently, the LDA topic containing the highest number of PASC testimonials was considered as the predicted Positive Class, while LDA topic containing the greatest number of non-PASC testimonials was considered as the predicted Negative Class. The same approach has been considered for topics extracted through a BERTopic-based approach. Then the accuracy was calculated by definition as:

$$\frac{TruePositive+TrueNegative}{187}$$

Table 2 show the topic distribution between the two classes for the LDA model. The number of PASC narratives belonging to Topic 2 has been considered as the number of True Positive, while the number of non-PASC texts assigned to Topic 1 has been considered as the number of True Negative.

Following the same approach for the BERTopic model, as reported in Table 2, the number of non-PASC documents assigned to Topic 1 has been considered as True Negative, while the number of PASC texts belonging to Topic 2 were considered as True Positive.

It is worth noting that both w.r.t. the LDA topic model and the BERTopic-based model, the topics with a prevalence of PASC were represented with 7 out of 10 common words, i.e., “fever”, “fatigue”, “tiredness”, “covid”, “tampon”, “smell”, “symptom” and “pain”, which refer to some typical symptoms of the PASC condition, while the topics with a prevalence of non-PASC have “patient” as the only word in common, but in general they contain more words related to the activity of medical personnel (“patient”, “collegues”, “intensive_care” etc.).

From Table 2 and Table 3, it follows that the LDA-based approach recognizes the 78.08% of the PASC narratives, reaching an overall accuracy of 86.10%, while the BERTtopic-based approach performs better with an accuracy of 91.97% and by assigning 97.26% of PASC narrations to the same cluster.

## 5. Conclusions

In the present study, LDA and a BERTopic-based methods have been proposed to characterize Italian narrative medicine texts written on COVID-19. In particular, the focus was mainly on the writings of patients with Post-acute sequelae of COVID-19, i.e., PASC, as opposed to writings by health professionals and general reflections on COVID-19, and tagged as non-PASC.

The results show that the BERTopic approach outperforms the LDA-base approach by grouping in the same cluster the 97.26% of texts, and reaching an overall accuracy of 91.97%.

The present study has shown that a characterization of PASC narration through topic modeling is feasible, and suggests that the testimonies of PASC patients needs to be further investigated for identifying shared issues to focus on in order to be followed and supported appropriately, even from a psychological point of view.

In this work we modeled the text of the generic patient with TM and SA tools, but in the future we could perform the text representation with TS, considering the text written by a patient at different times to evaluate the evolution of the disease over time. Moreover, we also plan to investigate the feasibility of the proposed method for prediction purposes, extending our approach to other COVID-19 datasets.

Finally, since HMMs have found application in many areas interested in NLP, it may be interesting to apply HMM or its AR-HMM or MOM extensions to Narrative Medicine and perhaps extend our long COVID study.

## Figures and Tables

**Table 1:** Performance of LDA wrt the number of topics. Best performance are reached by a number of topics equal to 3.

Number of Topics	Coherence Score	Perplexity
3	**0.407**	**−7.248**
5	0.332	−7.337
7	0.299	−7.398
10	0.346	−7.5239

**Table 2:** Distribution of LDA topics wrt the considered classes.

Assigned LDA Topic	Class	Document Count
1	non-PASC	104
	PASC	2
2	non-PASC	6
	PASC	57
3	non-PASC	4
	PASC	14

**Table 3:** Distribution of BERTopic topics wrt the considered classes.

Assigned BERTopic	Class	Document Count
−1	non-PASC	5
	PASC	1
1	non-PASC	101
	PASC	1
2	non-PASC	8
	PASC	71

## Data Availability

Publicly available data were analyzed in this study. Data can be found at “R-Esistere” https://www.medicinanarrativa.network/r-esistere/, accessed on 30 August 2022 and “Sindrome post COVID-19” https://www.sindromepostcovid19.it/, accessed on 30 August 2022.

## References

[1] Hossain M.M., Tasnim S., Sultana A., Faizah F., Mazumder H., Zou L., McKyer E.L.J., Ahmed H.U., Ma P. (2020). Epidemiology of mental health problems in COVID-19: A review. F1000Research.

[2] Rossi R., Socci V., Talevi D., Mensi S., Niolu C., Pacitti F., Di Marco A., Rossi A., Siracusano A., Di Lorenzo G. (2020). COVID-19 pandemic and lockdown measures impact on mental health among the general population in Italy. Front. Psychiatry.

[3] Maison D., Jaworska D., Adamczyk D., Affeltowicz D. (2021). The challenges arising from the COVID-19 pandemic and the way people deal with them. A qualitative longitudinal study. PLoS ONE.

[4] Wicke P., Bolognesi M.M. (2021). Covid-19 Discourse on Twitter: How the Topics, Sentiments, Subjectivity, and Figurative Frames Changed Over Time. Front. Commun..

[5] Chandrasekaran R., Mehta V., Valkunde T., Moustakas E. (2020). Topics, trends, and sentiments of tweets about the COVID-19 pandemic: Temporal infoveillance study. J. Med. Internet Res..

[6] Boon-Itt S., Skunkan Y. (2020). Public perception of the COVID-19 pandemic on Twitter: Sentiment analysis and topic modeling study. JMIR Public Health Surveill..

[7] Medford R.J., Saleh S.N., Sumarsono A., Perl T.M., Lehmann C.U. (2020). An “Infodemic”: Leveraging High-Volume Twitter Data to Understand Early Public Sentiment for the Coronavirus Disease 2019 Outbreak.

[8] Valdez D., Ten Thij M., Bathina K., Rutter L.A., Bollen J. (2020). Social media insights into US mental health during the COVID-19 pandemic: Longitudinal analysis of twitter data. J. Med. Internet Res..

[9] Zucco C., Calabrese B., Agapito G., Guzzi P.H., Cannataro M. (2020). Sentiment analysis for mining texts and social networks data: Methods and tools. Wiley Interdiscip. Rev. Data Min. Knowl. Discov..

[10] Rosenberg H., Syed S., Rezaie S. (2020). The Twitter pandemic: The critical role of Twitter in the dissemination of medical information and misinformation during the COVID-19 pandemic. Can. J. Emerg. Med..

[11] Jelodar H., Wang Y., Orji R., Huang S. (2020). Deep sentiment classification and topic discovery on novel coronavirus or COVID-19 online discussions: NLP using LSTM recurrent neural network approach. IEEE J. Biomed. Health Inform..

[12] Leung Y.T., Khalvati F. (2022). Exploring COVID-19 Related Stressors Using Topic Modeling. arXiv.

[13] Agrusta M., Cenci C. (2021). Telemedicine and digital narrative medicine for the customization of the diagnostic-therapeutic path at the time of COVID 19. JAMD.

[14] Hurwitz B., Cushing A., Chisnall B. (2012). Narrative medicine. BMJ.

[15] Mehandru S., Merad M. (2022). Pathological sequelae of long-haul COVID. Nat. Immunol..

[16] Taquet M., Luciano S., Geddes J.R., Harrison P.J. (2021). Bidirectional associations between COVID-19 and psychiatric disorder: Retrospective cohort studies of 62 354 COVID-19 cases in the USA. Lancet Psychiatry.

[17] Pye A., Roberts S.R., Blennerhassett A., Iqbal H., Beenstock J., Iqbal Z. (2021). A public health approach to estimating the need for long COVID services. J. Public Health.

[18] Baum L.E., Eagon J.A. (1967). An inequality with applications to statistical estimation for probabilistic functions of Markov processes and to a model for ecology. Bull. Am. Math. Soc..

[19] Baum L.E., Petrie T. (1966). Statistical inference for probabilistic functions of finite state Markov chains. Ann. Math. Stat..

[20] Blunsom P. (2004). Hidden markov models. Lect. Notes.

[21] Scarpino I., Zucco C., Cannataro M. Characterization of Long COVID using text mining on narrative medicine texts. Proceedings of the 2021 IEEE International Conference on Bioinformatics and Biomedicine (BIBM).

[22] Scarpino I., Zucco C., Cannataro M. (2021). A Software Pipeline Based on Sentiment Analysis to Analyze Narrative Medicine Texts. Proceedings of the International Conference on Computational Science.

[23] Devlin J., Chang M.W., Lee K., Toutanova K. (2018). Bert: Pre-training of deep bidirectional transformers for language understanding. arXiv.

[24] Lin T., Wang Y., Liu X., Qiu X. (2021). A survey of transformers. arXiv.

[25] Evans M. (2002). Reflections on the humanities in medical education. Med Educ..

[26] Charon R. (2008). Narrative Medicine: Honoring the Stories of Illness.

[27] Zannini L. (2008). Medical Humanities and Narrative Medicine: New Perspectives in Healthcare Professionals’ Training.

[28] Bernegger G., Castiglioni M., Garrino L. (2014). A doctor among clearings, tigers and jazz. A dialog with Rita Charon. J. Med Humanit..

[29] Owen W.F. (1984). Interpretive themes in relational communication. Q. J. Speech.

[30] Bakhtin M., Ghāsemipour G. (2011). The problem of speech genres. Lit. Crit..

[31] Weber R.P. (1990). Basic Content Analysis.

[32] Rushforth A., Ladds E., Wieringa S., Taylor S., Husain L., Greenhalgh T. (2021). Long Covid–The illness narratives. Soc. Sci. Med..

[33] Chyon F.A., Suman M.N.H., Fahim M.R.I., Ahmmed M.S. (2022). Time series analysis and predicting COVID-19 affected patients by ARIMA model using machine learning. J. Virol. Methods.

[34] Zhou S., Braca P., Marano S., Willett P., Millefiori L.M., Gaglione D., Pattipati K.R. (2021). Application of Hidden Markov Models to Analyze, Group and Visualize Spatio-Temporal COVID-19 Data. IEEE Access.

[35] Prabhu S.M., Subramaniam N. (2020). Surveillance of COVID-19 Pandemic using Hidden Markov Model. arXiv.

[36] Hearst M. (2003). What Is Text Mining.

[37] Sengupta S., Mugde S., Sharma G. (2020). An Exploration of Impact of COVID 19 on mental health-Analysis of tweets using Natural Language Processing techniques. medRxiv.

[38] Le Bras P., Gharavi A., Robb D.A., Vidal A.F., Padilla S., Chantler M.J. (2020). Visualising covid-19 research. arXiv.

[39] Älgå A., Eriksson O., Nordberg M. (2020). Analysis of scientific publications during the early phase of the COVID-19 pandemic: Topic modeling study. J. Med. Internet Res..

[40] Zengul F.D., Zengul A.G., Mugavero M., Oner N., Ozaydin B., Delen D., Willig J.H., Kennedy K.C., Cimino J. (2021). A critical analysis of COVID-19 research literature: Text mining approach. Intelligence-Based Med..

[41] Ghasiya P., Okamura K. (2021). Investigating COVID-19 News Across Four Nations: A Topic Modeling and Sentiment Analysis Approach. IEEE Access.

[42] Bai Y., Jia S., Chen L. (2020). Topic evolution analysis of COVID-19 news articles. J. Phys. Conf. Ser..

[43] Liu Q., Zheng Z., Zheng J., Chen Q., Liu G., Chen S., Chu B., Zhu H., Akinwunmi B., Huang J. (2020). Health communication through news media during the early stage of the COVID-19 outbreak in China: Digital topic modeling approach. J. Med. Internet Res..

[44] De Santis E., Martino A., Rizzi A. (2020). An infoveillance system for detecting and tracking relevant topics from Italian tweets during the COVID-19 event. IEEE Access.

[45] Noor S., Guo Y., Shah S.H.H., Fournier-Viger P., Nawaz M.S. (2020). Analysis of public reactions to the novel Coronavirus (COVID-19) outbreak on Twitter. Kybernetes.

[46] Han X., Wang J., Zhang M., Wang X. (2020). Using social media to mine and analyze public opinion related to COVID-19 in China. Int. J. Environ. Res. Public Health.

[47] Nguyen T.T., Nguyen Q.V.H., Nguyen D.T., Hsu E.B., Yang S., Eklund P. (2020). Artificial intelligence in the battle against coronavirus (COVID-19): A survey and future research directions. arXiv.

[48] Anderson B.S. (2021). Using text mining to glean insights from COVID-19 literature. J. Inf. Sci..

[49] Blei D.M., Ng A.Y., Jordan M.I. (2003). Latent dirichlet allocation. J. Mach. Learn. Res..

[50] Rehurek R., Sojka P. Software framework for topic modelling with large corpora. Proceedings of the LREC 2010 Workshop on New Challenges for NLP Frameworks.

[51] Vaswani A., Shazeer N., Parmar N., Uszkoreit J., Jones L., Gomez A.N., Kaiser Ł., Polosukhin I. (2017). Attention is all you need. Adv. Neural Inf. Process. Syst..

[52] Liu Y., Ott M., Goyal N., Du J., Joshi M., Chen D., Levy O., Lewis M., Zettlemoyer L., Stoyanov V. (2019). Roberta: A robustly optimized bert pretraining approach. arXiv.

[53] Sanh V., Debut L., Chaumond J., Wolf T. (2019). DistilBERT, a distilled version of BERT: Smaller, faster, cheaper and lighter. arXiv.

[54] McInnes L., Healy J., Melville J. (2018). Umap: Uniform manifold approximation and projection for dimension reduction. arXiv.

[55] McInnes L., Healy J., Astels S. (2017). hdbscan: Hierarchical density based clustering. J. Open Source Softw..

[56] Reimers N., Gurevych I. (2019). Sentence-BERT: Sentence Embeddings using Siamese BERT-Networks. Proceedings of the 2019 Conference on Empirical Methods in Natural Language Processing.

